# P285: Comparison of two nucleic acid amplification assays, the probetec et assay and xpert mtb/rif assay, for detection of mycobacterium tuberculosis in respiratory specimens

**DOI:** 10.1186/2047-2994-2-S1-P285

**Published:** 2013-06-20

**Authors:** S Aljohani, M Alshomrani

**Affiliations:** 1 Pathology and laboratory medicine, King Abdulaziz Medical City, Riyadh, Saudi Arabia; 2 Pathology and laboratory medicine, King Saud University, Riyadh, Saudi Arabia

## Introduction

Early diagnosis and management of TB is critical to reduce TB transmission in communities and health care facilities. In developing countries, TB diagnosis is largely based on smear microscopy of sputum samples.

Nucleic acid amplification tests (NAATs) have greatly facilitated TB testing by lowering the turnaround time to a day or less from two or more weeks for a culture result.

## Objectives

Our aim is to evaluate the diagnostic performance of the Xpert MTB/RIF and BD ProbTec Strand Displacement Amplification assays on smear-positive and smear-negative patient specimens submitted to King Abduaziz Medical City laboratory, in Riyadh, Saudi Arabia from July to Mid Nov 2012, the study still ongoing to collect more data.

## Methods

A total of 35 consecutive specimens were subjected to the two molecular assays, and their performance characteristics were assessed relative to the routine diagnostic standard. Both assays showed similar diagnostic performance characteristics.

Clinical specimens were divided into two equal portions, one for the Xpert MTB/RIF test and the other one for ProbTec ET assay. NAAs were applied in parallel in a blind manner by one laboratory technician, and the results from the two assays were compared by a second person.

The Xpert MTB/RIF method BD ProbeTec performed as per manufacturer instructions.

## Results

Of the 35 samples tested, there were seven samples that are positive by both PCR as well as by culture. Eight samples were positive by ProbTec alone and one sample positive by Xpert alone. The sensitivities of the BD ProbTec and Xpert MTB/RIF assays for the detection of culture-positive M. tuberculosis were 87.5%, while the specificities of both assays were 70%> 96.3% , respectively. Both assays were able to diagnose the presence of M. tuberculosis in 57 to 58% of smear-negative cases, suggesting that the performance characteristics were dependent on bacillary load.

## Conclusion

Performance of both assays relatively good, Performance of GeneXpert superior to BD probTec, inconjuction to ease of use and availability of Rifampin susceptibility result makes it promising tool to test direct patient's sample.

## Competing interests

None declared

